# Influence of Different Metal Types on the Bonding Strength of Concrete Using the Arc Thermal Metal Spraying Method

**DOI:** 10.3390/ma16072651

**Published:** 2023-03-27

**Authors:** Jin-Ho Park, Sang-Youl Kim, Han-Seung Lee, Kwangwoo Wi

**Affiliations:** 1 Department of Research Promotion Team, Hanyang University, 1271 Sa 3-dong, Sangrok-gu, Ansan 15588, Republic of Korea; 2 Department of Architectural Engineering, Hanyang University, 1271 Sa 3-dong, Sangrok-gu, Ansan 15588, Republic of Korea; 3 Innovative Durable Building and Infrastructure Research Center, Hanyang University, 1271 Sa3-dong, Sangrok-gu, Ansan 15588, Republic of Korea

**Keywords:** metals, arc thermal metal spraying, bonding strength, non-interfacial failure

## Abstract

Exterior finishes protect reinforced concrete buildings against environmental factors, improve their durability, and enhance their exterior design. In this study, the influence of different metal types used in arc thermal metal spraying on the adhesion between concrete and metal coatings was analyzed. Five metals with different melting points were tested, and the differences between their melting points and surface temperatures immediately after thermal spraying were measured. The bonding strength of each metal was evaluated. Additionally, the interface between the concrete surface and metal coating was analyzed using image analysis and optical microscopy. The results demonstrated that Zn achieved the highest bonding strength (1.84 MPa), which had the lowest melting point and surface temperature immediately after spraying, while Cu/Sn achieved the lowest strength (1.38 MPa), which had the highest temperatures. The bonding strength had a closer relationship (R^2^ = 0.9946) with the difference between the melting point and surface temperature immediately after spraying than that (R^2^ = 0.9589) with the surface temperature immediately after spraying. The bonding strength increased as the ratio of the non-interfacial failure area to the total area increased, ensuring a stronger attachment to the concrete surface. Overall, the results showed that the bonding strength was significantly affected by the metal type.

## 1. Introduction

The exterior finishes of reinforced concrete buildings provide an important protective element against environmental factors, such as temperature, snow, wind, rain, and external disasters. Furthermore, they can improve the durability and exterior design of buildings. Therefore, sufficient considerations are required for appropriately selecting the exterior finishing materials and methods [1,2,3].

The finishing methods currently applied to building structures include the wet method of directly applying paint and plaster materials to the structure and the dry method of attaching stone materials and panels [4]. The wet finishing method is characterized by simple construction and low initial construction costs. Still, it requires high maintenance costs because the maintenance interval is short, owing to surface contamination and low weather resistance. Moreover, after re-coating, the coating layer becomes thicker, thereby decreasing the bonding performance. Conversely, the dry finishing method ensures high durability but involves difficult maintenance and repair processes due to damage to finishing materials and construction difficulties associated with anchor treatment [3,4,5,6]. To overcome the shortcomings of conventional finishing methods (wet and dry methods), methods that simultaneously serve as a structure and finish material, such as exposed concrete, have been developed. In such methods, cracks propagate in the finished area if the initial construction of the structure is not performed properly, and the durability rapidly decreases without appropriate surface maintenance. The exposed concrete also has limitations in expressing the outer wall color and three-dimensional design of the building. As such, the development of a finishing method that involves simple construction and excellent building design while retaining the benefits of the dry method is required, which uses highly durable metal panels [3,6,7].

Recently, metal-film finishing methods have been developed to apply arc thermal metal spraying (ATMS) to water treatment and electromagnetic wave shielding facilities [6,8,9,10,11]. These new finishing methods apply metal spraying, a conventional technology mainly used in high-durability anti-corrosion applications for steel structures [5,10]. The anti-corrosion lifespan of ATMS is up to approximately 100 years, depending on the metal type, and is approximately three to four times longer than that of the paints used in the wet method [5,12]. These methods involve almost no defects because quality control can be performed during construction. They have high maintenance efficiency because only the damaged section must be repaired in the case of local damage [5,6,8,10,13,14,15]. However, the bond performance between the metal film and substrate is expected to vary significantly depending on the substrate conditions and type of metal-film finishing material [3,5,10,11]. In particular, when a metal film is directly applied to a concrete structure, the bond performance between the metal film and concrete is expected to vary depending on the metal because of property changes, such as the melting point. In previous studies, however, only the surface treatment of concrete, which is the substrate, and change in bonding strength according to moisture conditions have been considered [6]. No studies have been conducted on the influence of the metal type on the bonding strength [5,8,10].

Therefore, this study aims to evaluate the influence of the metal used as a wire material in ATMS on the concrete bonding strength. Metal coatings were formed on the concrete surface using various metals. The surface temperature of the coatings was measured immediately after spraying, as well as the bonding strength. In addition, the interface between the concrete surface and metal coating was evaluated through image analysis of the fracture sections and optical microscopy (OM).

## 2. Experimental Program

### 2.1. Experimental Overview

In this study, various experiments were performed to examine the bonding strength between the metal coating and concrete based on the metal type. Table 1 lists the experimental levels, factors, and items considered in this study.

Typically, concrete with a strength of 27 MPa is used as the substrate. The most efficient surface treatment methods in terms of the bonding strength determined in previous studies (sand blasting → surface hardener (SH) → surface roughness agent (SRA)) were used in this study [2,3]. In the case of sand blasting, silica sands with a particle size of 1.2–1.6 mm were blasted onto the concrete surface using high-pressured air to clean up the concrete surface and achieve a basic roughness.

After surface treatment, metal spraying was performed using five metals. A commonly used metal spray coating thickness of 200 μm was adopted [16]. The bonding strength and failure mode were evaluated as experimental parameters. The temperature of the metal applied to the surface was measured immediately after the spraying. Image analysis was conducted on fracture sections, and the concrete cross-section subjected to metal spraying was observed using a microscope.

### 2.2. Metals

For the appropriate application of ATMS to concrete surfaces, metals with a low melting point, unique color, and high durability are chosen from the metals that can be sprayed [2,3,4]. Therefore, in this study, Zn (SAMHWA NON-FERROUS METAL IND, Seoul, Republic of Korea), Al (SAMHWA NON-FERROUS METAL IND, Republic of Korea), and a Zn/Al alloy (SAMHWA NON-FERROUS METAL IND, Republic of Korea), which are mainly used in anti-corrosion techniques for steel structures, were used. Brass (Cu/Zn, KORYEO, China) and bronze (Cu/Sn, KORYEO, China), which have excellent color and metal-spraying efficiency with high corrosion resistance, were also used.

Table 2 lists the properties of the metals used in this study. For Zn, 100% pure white Zn with a melting point of 420 °C and density of 7.13 g/cm^3^ was used. For Al, 100% pure white Al with a melting point of 660 °C and density of 2.70 g/cm^3^ was used. In the case of the Zn/Al alloy, a gray alloy (Zn 85% and Al 15%) with a melting point of 540 °C and density of 4.91 g/cm^3^ was used in the experiment. Cu/Zn and Cu/Sn with melting points of 914 and 1018 °C, densities of 8.60 and 8.75 g/cm^3^, and metal ratios of 8:2 and 9:1, respectively, were used.

### 2.3. Fabrication of Concrete as a Substrate

The cement used in the substrate concrete was ordinary Portland cement (Asia cement, Seoul, Republic of Korea), adhering to the KS L 5201 regulations [17]. Table 3 and Table 4 list the chemical composition and physical properties of the cement used. Table 5 lists the mixing proportions of the concretes used in this study. The water-to-binder (W/B) ratio was set to 0.5. Ordinary crushed stone with a maximum size of 25 mm (specific gravity of 2.64 and water absorption of 0.77%) was used for the coarse aggregate, and sea sand with a fineness modulus of 2.9 (specific gravity of 2.59) was used for the fine aggregate. In addition, a polycarboxylate superplasticizer (HMS, Republic of Korea) was used to improve the workability of concrete. Immediately after mixing, the concrete was poured into a 300 mm × 300 mm × 50 mm mold and compacted using a vibration table. The specimen was left in the mold for one day and subjected to water curing for 27 days. The strength [18], slump [19], and air content [20] of the cured concrete were evaluated according to the KS standards; the results are listed in Table 6.

### 2.4. ATMS

**Figure 1:**
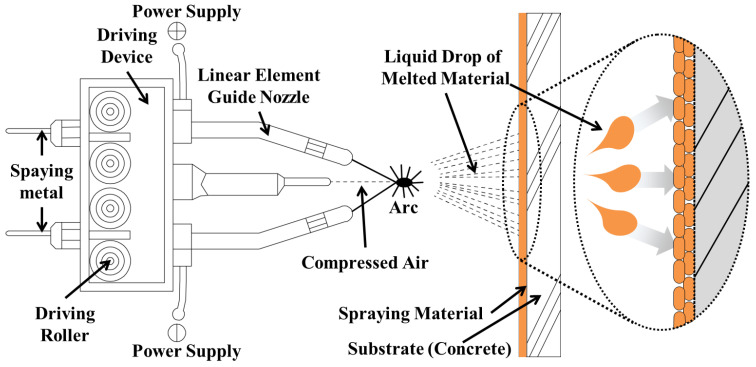
Schematic of ATMS implementation.

ATMS is a technology that forms a porous, strong, and stable film on the material surface by melting wire or powder metals using gas or electric arcs and spraying the molten metals under a high-pressure environment with six bars or higher [21,22,23,24]. ATMS can be categorized into gas and electrical spraying, according to the heat source. Because gas metal spraying has certain safety concerns owing to using a gas as the heat source, the experiments in this study were performed using high-frequency arc thermal metal spraying, which is an electrical spraying technique, as shown in Figure 1. High-frequency arc thermal metal spraying enables a safe construction, relatively high lamination speed, and a wide range of coatings [5,21,24].

**Figure 2:**
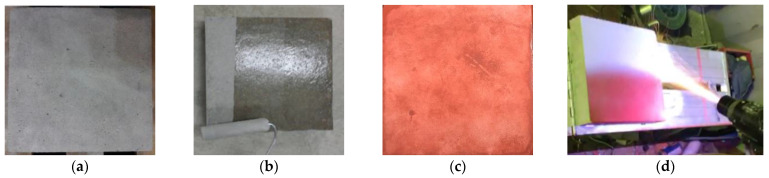
Procedure of ATMS. (**a**) Cleaning surface with sand blasting; (**b**) Surface hardener (SH); (**c**) Surface roughness agent (SRA); (**d**) ATMS.

The concrete cured for 28 days was dried at 100 °C for two days and subjected to sand blasting for surface cleaning. Subsequently, SH (TAS, Republic of Korea) was applied to increase the concrete surface strength, which is expected to affect the bonding strength as well [3,25]. SRA (EMS, Republic of Korea) was then applied to increase the roughness of the concrete surface. According to the experimental program, five metals were applied with a thickness of 200 μm. To ensure that metal spraying coatings with a constant thickness were obtained, the coating thickness was measured using a Vernier caliper after the operation. Figure 2 shows the specimen preparation process, and Table 7 lists the physical properties of the SH and SRA used in this study.

### 2.5. Test Methods

#### 2.5.1. Temperature Measurement

**Figure 3:**
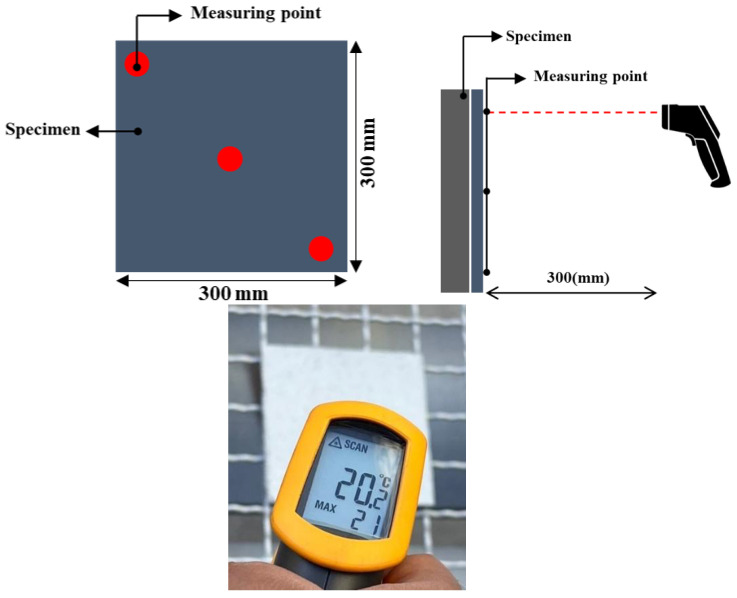
Temperature measurement overview.

Immediately after metal spraying, the surface temperature was measured using an infrared thermometer (FLUKE-62 Mini) on three points at a distance of 300 mm, as shown in Figure 3. Table 8 lists the basic technical specifications of the thermometer. In this study, the three measurements were averaged to evaluate the overall surface temperature. At the time of measurement, the ambient temperature in the laboratory was adjusted to 20 ± 2 °C to minimize the influence of the external temperature.

#### 2.5.2. Bonding Strength and Failure Mode

**Figure 4:**
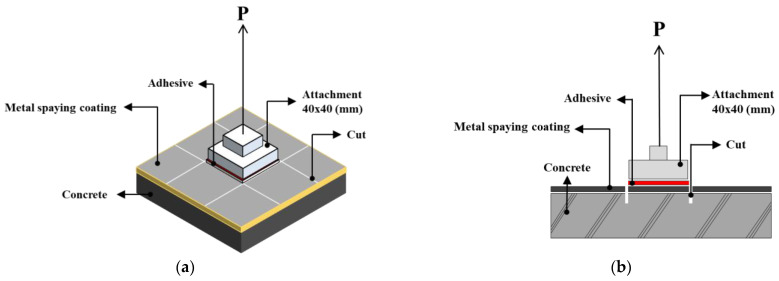
Schematic of the bonding strength measurement. (**a**) Top view; (**b**) Side view.

The bonding strength was evaluated according to the KS F 4716 [26] and ASTM D 4541 [27]. As shown in Figure 4, a 40 mm × 40 mm square tensile attachment was adhered to the specimen with a metal spraying coating for each experiment using an epoxy adhesive and dried for 24 h. A vertical cut was made on the concrete surface around the attachment, as shown in Figure 4b. The bonding strength was calculated using Equation (1) after measuring the maximum load applied during the tensile bond test. The average of nine bonding strength measurements was evaluated as the overall bonding strength. Thereafter, the bond failure behavior of the metal film was visually observed, and an image analysis was conducted.

(1) $$Bonding\;Strength\;\left({N/mm}^{2}\right)=\frac{Maximum\;tensile\;load\;(N)}{Attachment\;area\;(1600\;{mm}^{2})}$$

#### 2.5.3. Image Analysis

**Figure 5:**
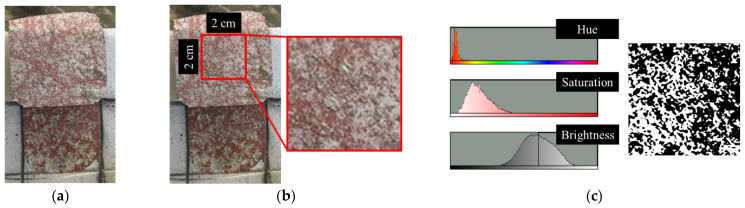
Procedure of image analysis using ImageJ. (**a**) Original image; (**b**) Area detection for image analysis; (**c**) Color thresholding.

After the bonding strength measurements were completed, image analysis was conducted on the fracture sections to examine the failure pattern between the metal spraying coating and concrete surface and quantitatively calculate the non-interfacial failure area. Figure 5 shows the image analysis procedure adopted in this study to calculate the area quantitatively. ImageJ software was used for image analysis [28]. After completing the bonding strength measurements, images of the fracture section were captured using a high-definition camera (Figure 5a). Thereafter, a 2 cm × 2 cm area was set in the center of the fracture section as the measurement area (Figure 5b). Because the SRA used in this study was red, the area that did not exhibit a red color was quantitatively calculated using the color thresholding function (Figure 5c). In the case of non-interfacial failure, concrete failure occurs rather than failure between the metal spraying coating and concrete surface coated with SRA. Therefore, the areas that did not exhibit a red color were quantitatively measured. Finally, the ratio of the non-interfacial failure area to the total area (RN) was calculated using the following equation:

(2) $$RN\left(\%\right)=\frac{Non-interfacial\;failure\;area}{Total\;area}\times 100$$

#### 2.5.4. OM

**Figure 6:**
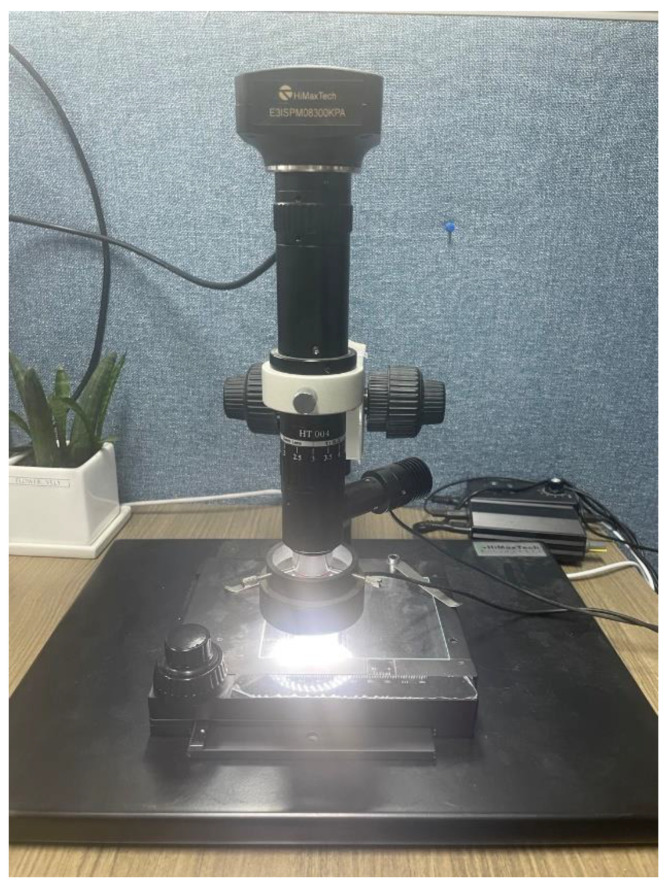
Optical microscope used for OM.

An OM (HT004, HiMaxTech) was used to observe the cross-sectional area of the specimen, as shown in Figure 6. To minimize the damage inflicted on the concrete surface and metal coating, OM samples were prepared in two steps. First, samples of 4 cm × 4 cm × 2 cm were cut using a sawing machine, and samples of 1 cm × 1 cm × 0.5 cm were prepared using a diamond cutting machine. The samples were then immersed in low-viscosity epoxy for 24 h. Once the epoxy was completely hardened, the cut surfaces of the samples were polished using a polishing machine (EcoMet 30) in the order of P400 -> P2400 -> P4000 -> 3 μm -> 1 μm using a SiC paper and IPA solution. The SiC paper was first washed in an ultrasonic bath before being replaced to prevent contamination by impurities on the cut surface.

## 3. Results and Discussions

### 3.1. Temperature

**Figure 7:**
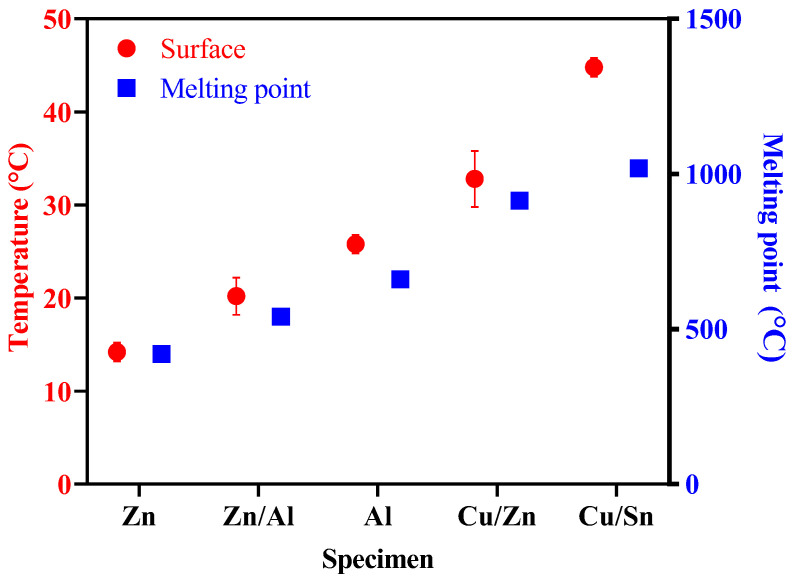
Surface temperature and melting point.

Figure 7 shows the melting points of the metals used in this study and their surface temperatures immediately after spraying. Zn exhibited the lowest surface temperature of approximately 14.2 °C, whereas Cu/Sn exhibited the highest surface temperature of approximately 44.8 °C. For the sprayed metals, the surface temperature increased in the order of Zn (14.2 °C), Zn/Al (20.2 °C), Al (25.8 °C), Cu/Zn (32.8 °C), and Cu/Sn (44.8 °C), indicating that the surface temperature was higher for metals with higher melting points.

**Figure 8:**
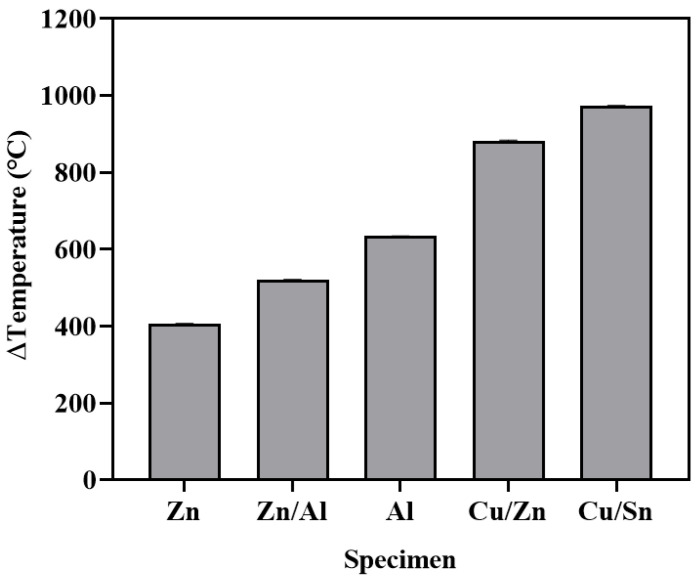
Difference between surface temperature and melting point.

Figure 8 shows the difference between the melting point and surface temperature immediately after spraying for each metal. Cu/Sn exhibited the largest difference (approximately 973.2 °C), followed by Cu/Zn (881.2 °C), Al (634.2 °C), Zn/Al (519.8 °C), and Zn (405.8 °C). ATMS is a technology that applies a coating to a surface by instantly melting a metal wire heated using electric arcs, and then spraying the molten metal using compressed air under six bars or higher. The metal temperature at the concrete surface was significantly lower than its melting point because the metal wire that was melted by the arc point was cooled by compressed air [21,22,23,24].

### 3.2. Bonding Strength

**Figure 9:**
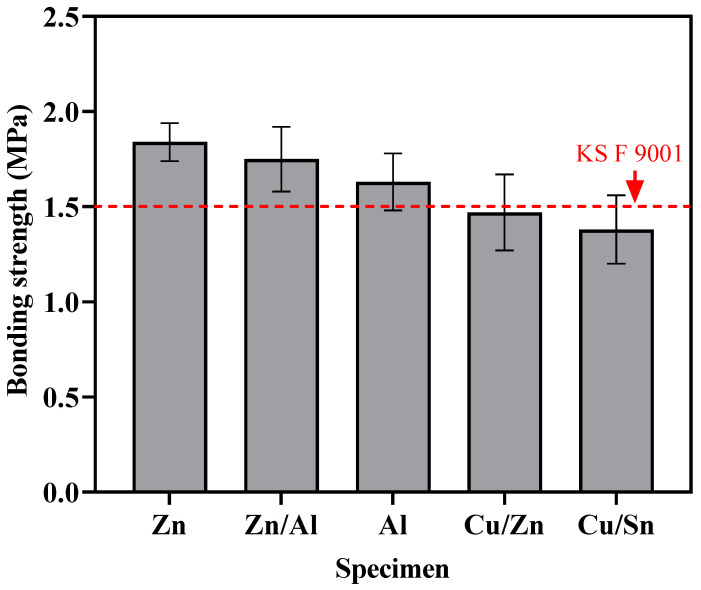
Results of bonding strength tests.

Figure 9 shows the results of the bonding-strength evaluation for each metal. Zn exhibited the highest bond performance of approximately 1.84 MPa. Zn/Al and Al showed bonding strengths of approximately 1.75 and 1.63 MPa, respectively. These results exceed the performance criterion of KS F 9001 (1.5 MPa). However, it was found that Cu/Zn and Cu/Sn, with bonding strengths of 1.47 and 1.38 MPa, respectively, did not meet the criterion.

**Figure 10:**
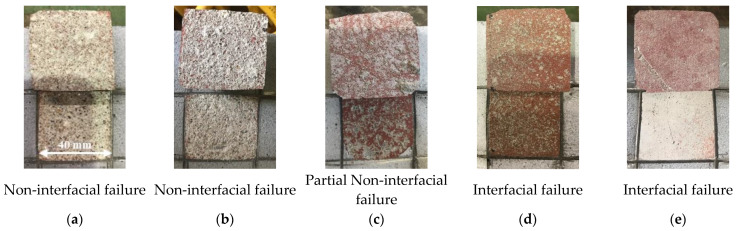
Failure modes. (**a**) Zinc; (**b**) Zinc/Al; (**c**) Al; (**d**) Cu/Zn; (**e**) Cu/Sn.

Visual inspection of the failure pattern revealed that the non-interfacial or partial non-interfacial failures of Zn, Zn/Al, and Al met the performance criterion of KS F 9001, as shown in Figure 10. In other words, concrete failure (non-interfacial failure) was dominant rather than failure at the interface between the metal spraying coating and concrete surface (interfacial failure). Conversely, the interfacial failures of Cu/Zn and Cu/Sn did not meet the performance criterion. Therefore, within the scope of this study, non-interfacial failure was determined to occur at approximately 1.63 MPa based on the bonding strength.

**Figure 11:**
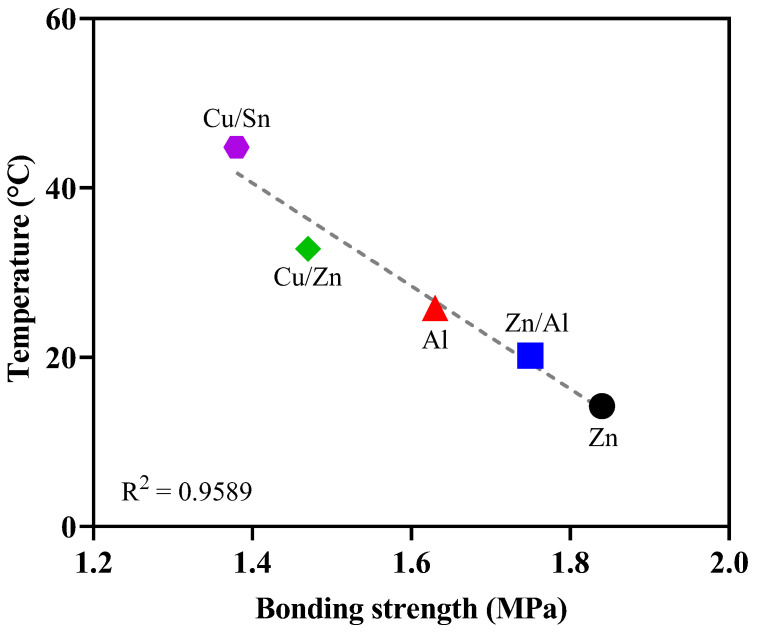
Relationship between the bonding strength and surface temperature.

**Figure 12:**
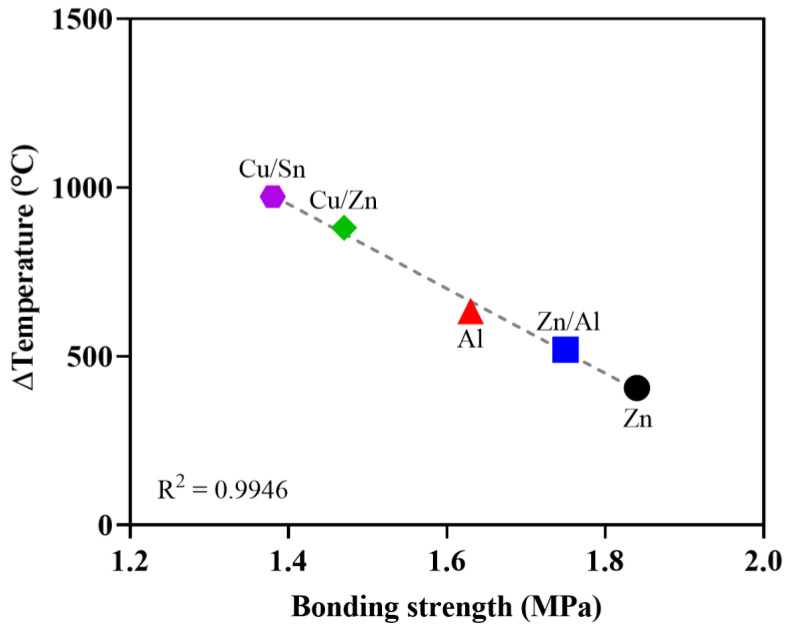
Relationship between the bonding strength and Δtemperature.

Figure 11 shows the correlation between the surface temperature and bonding strength when metals were sprayed. Figure 12 shows the correlation between the bonding strength and the difference between the melting point and surface temperature. The bonding strength decreased as the surface temperature increased immediately after metal spraying. Moreover, an increase in the surface temperature immediately after metal spraying had a negative impact on the bonding strength. Similarly, the bonding strength decreased as the difference between the melting point and surface temperature increased immediately after metal spraying. Because the bonding strength was found to have a closer correlation (R^2^ = 0.9946) with the difference between the melting point and surface temperature immediately after metal spraying compared to its correlation (R^2^ = 0.9589) with the surface temperature immediately after metal spraying, the difference between the melting point and surface temperature immediately after metal spraying was assessed to have a more significant effect on the bonding strength. Voids are generated in the metal spraying coating because of the shrinkage between the metal particles as the sprayed metal rapidly cools. Furthermore, the bonding strength is decreased by the voids between the concrete surface and metal spraying coating [29].

### 3.3. Image Analysis

**Figure 13:**
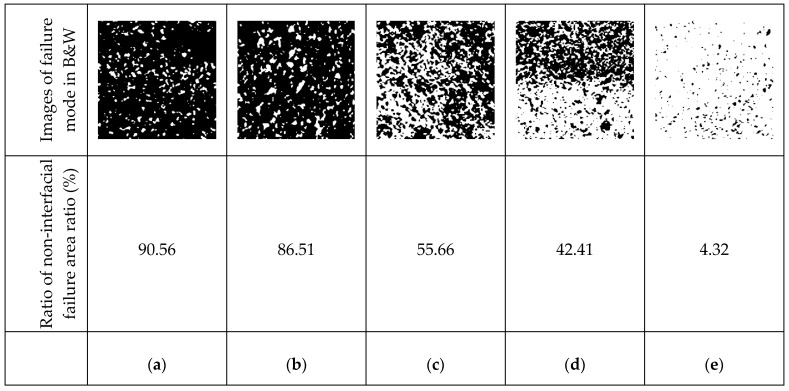
Ratio of non-interfacial failure area to total area. (**a**) Zn; (**b**) Zn/Al; (**c**) Al; (**d**) Cu/Zn; (**e**) Cu/Sn.

Figure 13 shows the interfacial and non-interfacial failure areas of the samples in black and white. RN results were calculated using image analysis. The RN values for Zn, Zn/Al, Al, Cu/Zn, and Cu/Sn were 90.56%, 86.51%, 55.66%, 42.41%, and 4.32%, respectively. Zn exhibited the highest RN value. This indicates that non-interfacial failure was dominant in the failure area. Moreover, Zn exhibited the highest bonding strength because non-interfacial failure was dominant. In contrast, Cu/Sn exhibited the lowest RN value, which indicates that interfacial failure was dominant compared to non-interfacial failure. The interfacial failure is judged to have a negative impact on the bonding strength rather than the concrete failure itself.

**Figure 14:**
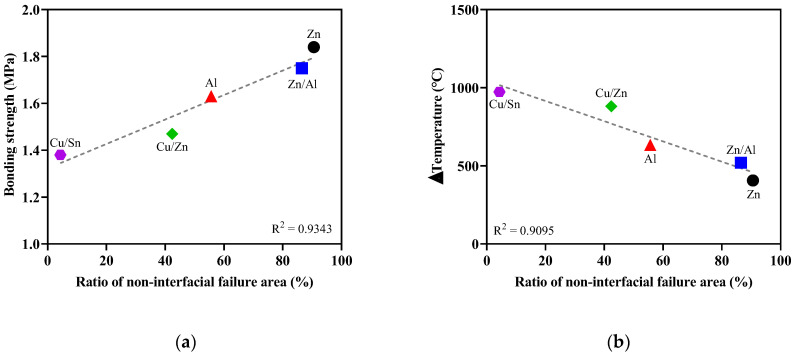
Relationship RN and (**a**) bonding strength and (**b**) Δtemperature.

Figure 14 shows the correlations between RN and bonding strength and temperature difference. As the RN value increased, the bonding strength of the samples also increased. This indicates that the bonding strength increased because non-interfacial failure was more dominant than interfacial failure. Conversely, as shown in Figure 14b, the difference between the melting point and surface temperature immediately after metal spraying tended to decrease as the RN value increased. The temperature difference and bonding strength showed a close correlation (R^2^ = 0.99), as mentioned in the previous section. In other words, the significant difference between the melting point of the metal and surface temperature immediately after metal spraying had a negative impact on the bonding strength because it induced more failures at the interface between the concrete surface and metal coating (interfacial failure) than that of the concrete (non-interfacial failure).

### 3.4. OM

**Figure 15:**
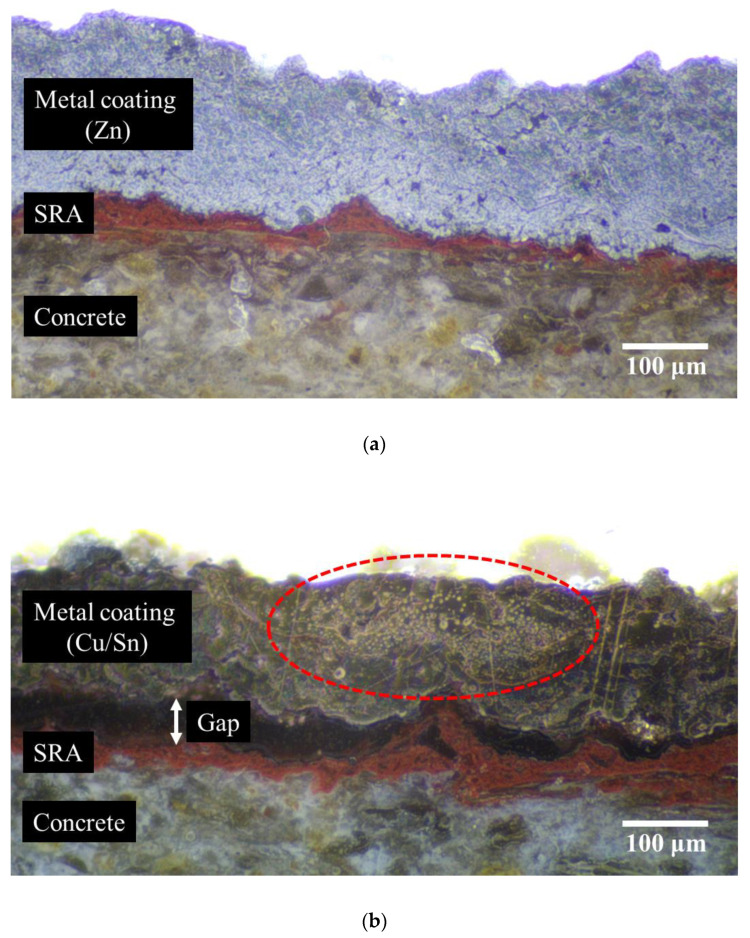
Cross-sectional areas of specimens. (**a**) Zn; (**b**) Cu + Sn.

Figure 15 shows the cross-sections of Zn, which exhibited the highest bonding strength, and Cu/Sn, which showed the lowest bonding strength. The application of SRA to the concrete surface, followed by ATMS using different metals while preparing the specimens, allowed the red epoxy (SRA) between the metal coating and the concrete surface to be observed in the two cross-sectional images.

As shown in Figure 15a, the concrete and SRA were in close contact, and the SRA was closely attached to the metal coating in the Zn specimen. Conversely, in the case of Cu/Sn, the concrete and SRA were in close contact, as shown in Figure 15b; however, the metal coating and SRA were not closely attached to each other, which confirmed the gap between them.

Concrete surface treatment was conducted for the application of ATMS (SHA and SRA were applied). Metal coatings were generated on the concrete surface by applying ATMS. In this instance, only the metal type differed, whereas the distance between the concrete and metal spraying gun (300 mm) and the ambient temperature (20 ± 2 °C) were kept constant. Therefore, the adhesion between the concrete surface and metal coating was only affected by the metal type. During the application of ATMS, rapid cooling occurred because of the temperature difference between the ambient environment and metal particles when the metal particles collided with the concrete surface, causing the deposited metal particles to contract [29]. Therefore, the amount of contraction is expected to be larger for metals with high melting points than those with low melting points because of the larger temperature difference. For this reason, Cu/Sn, with the highest melting point, could not develop sufficient cohesiveness with SRA.

In addition, a close look at the metal coating revealed that the metal coating with Zn had a more compact cross-sectional geometry than that with Cu/Sn. In particular, voids of various sizes are observed in the red dotted circle depicted in Figure 15b. This indicates that voids were generated between the metal particles because adhesion between the metal particles and adhesion between the SRA and metal was not sufficient owing to contraction, as mentioned above.

## 4. Conclusions

1. The surface temperature varied depending on the type of metal used. Zn showed the lowest surface temperature, while Cu/Sn exhibited the highest. The surface temperature immediately after metal spraying increased with the metal’s melting point.
2. The strength of the bond between the concrete and metal coatings also differed depending on the type of metal used. Zn exhibited the highest bonding strength (1.84 MPa), while Cu/Sn showed the lowest (1.38 MPa). Upon examining the failure modes, non-interfacial failure occurred mostly with Zn, whereas interfacial failure occurred mostly with Cu/Sn.
3. Because the bonding strength exhibited a close relationship (R^2^ = 0.9946) with the difference between the melting point of the metal and surface temperature immediately after metal spraying compared to its relationship (R^2^ = 0.9589) with the surface temperature immediately after metal spraying, the difference between the melting point and surface temperature immediately after metal spraying was assessed to have a greater impact on the bonding strength between the sprayed metal coating and concrete.
4. The non-interfacial failure area was quantitatively evaluated using image analysis, and the results exhibited a tendency similar to the bonding strength. Zn exhibited the highest ratio of the non-interfacial failure area to total area (RN), whereas Cu/Sn exhibited the lowest RN value.
5. The metal coating was found to be closely attached to the concrete surface with Zn; however, there was a gap between the metal coating and the concrete surface with Cu/Sn. This gap induces interfacial failure and ultimately has a negative impact on the bonding strength.
6. The metal type (namely, the melting point of the metal) was found to have a significant influence on the bonding strength. For the application of metals with a high melting point to the concrete surface, further research on surface treatment methods is required to ensure adequate bonding strength.

## Figures and Tables

**Table 1:** Experimental factors and measuring catalog.

Experimental Factor	Experimental Level	Measure Catalog
Types of metals	Zn	Temperature measurement
	Al	Bonding strength
	Zn/Al	Failure mode analysis
	Cu/Zn (Brass)	Image analysis
	Cu/Sn (Bronze)	Optical microscopy
Common list	Substrate: Concrete Concrete Strength: 27 MPa Concrete Size: 300 mm × 300 mm × 50 mm Surface treatment: (1) Sand blasting → (2) SH → (3) SRA Coating Thickness: 200 μm	

**Table 2:** Properties of metals used in ATMS.

Metal	Melting Point (°C)	Density (g/cm^3^)	Weight Ratio (%)	Color
Zn	420	7.13	100	Light gray
Zn/Al	540	4.91	85/15 (Zn/Al)	Gray
Al	660	2.70	100	White
Cu/Zn	914	8.60	80/20 (Cu/Zn)	Red brown
Cu/Sn	1018	8.75	90/10 (Cu/Sn)	Red gold

**Table 3:** Chemical composition of the cement used in this study.

	SiO_2_ (%)	Al_2_O_3_ (%)	Fe_2_O_3_ (%)	CaO (%)	MgO (%)	Na_2_O (%)	K_2_O (%)	SO_3_ (%)	LOI
Cement	19.5	5.2	2.7	61.8	3.7	2.5	0.1	0.8	2.6

**Table 4:** Physical properties of the cement used in this study.

	Density (g/cm^3^)	Blaine (cm^2^/g)	44 μm on Residue (%)	Setting Time (min)		Compressive Strength (MPa)		
				Initial	Final	3 Day	7 Day	28 Day
Cement	3.14	3200	12.5	240	370	22.5	30.0	39.5

**Table 5:** Mix proportion of the concrete used in this study.

Specimen	W/B	Unit Weight (kg/m^3^)				Superplasticizer (%)
		Water	Cement	Fine Aggregate	Coarse Aggregate	
Concrete	0.5	175	350	905	835	0.8

**Table 6:** Physical properties of the concrete used in this study.

Specimen	Slump (mm)	Air content (%)	7D-Compressive Strength (MPa)	28D-Compressive Strength (MPa)
Concrete	180	4.5	20	27

**Table 7:** Physical properties of SH and SRA.

Type	Element	Density (g/m^3^)	Usage (g/m^2^)
Surface hardener (SH)	Silicate	1.10	500–700
Surface roughness agent (SRA)	Epoxy, silica	1.30	50

**Table 8:** Properties of the infrared thermometer used in this study.

Temperature Range (°C)	Distance: Spot Size	Accuracy (°C)	Emissivity	Response Time (ms)
−30–500 °C	10:0.1	±1	0.95	<500

## Data Availability

Not applicable.
